# Analysis of Soft Tissue Changes and Influencing Factors of Implant Absorption after Immediate Restoration of Anterior Teeth

**DOI:** 10.1155/2022/3759337

**Published:** 2022-07-04

**Authors:** Guodong Peng, Xiaodong Sun, Xiang Xu

**Affiliations:** ^1^Department of Stomatology, The First People's Hospital of Jingzhou, Jingzhou, Hubei 434000, China; ^2^Department of Stomatology, Yichang Central People's Hospital, Yichang, Hubei 443003, China

## Abstract

**Objective:**

Using a digital model, evaluate the changes in the soft tissue following rapid restoration of anterior teeth and analyze the factors impacting implant absorption.

**Methods:**

A retrospective analysis was performed on 84 patients who received immediate implant restoration for a single anterior tooth in the department of Stomatology of our hospital from April 2020 to August 2021. According to different surgical methods, they were divided into the study group (*n* = 42) and control group (*n* = 42). Immediate implant repair was given to the research group, while delayed implant restoration was given to the control group. The influence of the two surgical techniques on the alterations of soft tissues around implants was studied using a 3Shape oral scan and a digital model before and 1, 3, and 5 months after the operation, respectively. Patients in the study group were divided into the excellent group (*n* = 26) and poor group (*n* = 16) according to the test results of implant bone absorption, and the risk factors of poor implant absorption after immediate restoration of anterior teeth were analyzed by univariate and multivariate analyses.

**Results:**

The levels of 1 mm and 3 mm below the gum mucosa margin in the two groups increased gradually with the time, and the gingival level and soft tissue thickness at the lip of the baseline implant also increased gradually. However, the changes of soft tissue in the study group were better than those in the control group at 3 and 6 months after surgery (*P* < 0.05). The PES score was significantly improved in both groups after treatment, and the aesthetic score was higher in the study group than in the control group (*P* < 0.05). Univariate and binary logistic multifactor regression showed that smoking and poor implant health were the related factors affecting implant absorption (*P* < 0.05).

**Conclusion:**

Immediate anterior tooth implantation and pharyngeal implant restoration can better restore the soft tissue and aesthetic degree of patients, but immediate implant restoration can more effectively restore the soft tissue, and controlling smoking and keeping clean around the implant after surgery is conducive to implant absorption.

## 1. Introduction

Oral implant repair is the main method for the treatment of dentition defect and dentition loss. For patients with dentition loss in the aesthetic area of front teeth, traditional implant treatment will increase the repair time of missing teeth due to the prolonged operation time, which will have varying degrees of impact on the quality of life and emotion of patients [1]. With the advancement of medical technology, immediate anterior tooth implant repair has become more extensively used in clinical practice to obtain better aesthetic results by reducing the time it takes for missing teeth to recover [2]. However, immediate implantation still has some limitations. Some studies have pointed out that the potential keratinized mucosa defect in the surgical area will reduce the initial stability of the implant, and the surface soft tissue morphology of the alveolar bone will also change with tooth extraction [3, 4]. Therefore, the evaluation of soft tissue morphology has always been a difficult problem in clinical practice. Some scholars used plaster model to measure and evaluate oral soft tissue before, but the results produced errors due to the shrinkage and expansion of model materials [5]. However, because the continuous development of three-dimensional image technology provides a technical basis for soft tissue evaluation, this study created a digital model to observe the impact of different anterior tooth restoration implant methods on patient soft tissue changes and then used a multifactor analysis to identify risk factors for poor implant absorption after surgery. To provide a more effective treatment for patients with dentition defect in the aesthetic area of anterior teeth and lay a theoretical foundation for improving prognosis, it will be reported as follows [6].

The arrangements of this paper are as follows: Section 1 discusses the general information and methods.  Section 2 examines an experimental result.  Section 3 concludes the article with discussion.

## 2. General Information and Methods

### 2.1. General Information

A retrospective analysis was performed on 84 patients who underwent immediate implant restoration of the single anterior tooth in stomatology Department of our hospital from April 2020 to August 2021. According to different surgical methods, they were divided into the study group (*n* = 42) and control group (*n* = 42). The comparison of baseline data between the two groups was shown in Table 1, with no significant difference (*P* > 0.05). In addition, patients in the study group were divided into the excellent group (*n* = 26) and poor group (*n* = 16) according to the test results of bone absorption of implants. Before surgery, all patients in the study completed an informed consent form, and the general information and clinical data gathered in this study were kept private and not utilized for any other reason.

Inclusion criteria are as follows: (1) patients did not have inflammation at the root tip, (2) the patient had a healthy gingiva and stable occlusal relationship, (3) no contraindications of other dental implants;, and (4) signed informed consent.

Exclusion criteria are as follows: (1) patients with mental diseases cannot cooperate with the study, (2) unclear clinical imaging data, (3) patients with poor treatment compliance, which influenced the study results, and (4) combined with autoimmune dysfunction.

### 2.2. Methods

#### 2.2.1. Treatment Methods

Before treatment, both groups were given the same routine treatment such as periodontal cleaning and local anesthesia during the operation.

**Figure 1:**
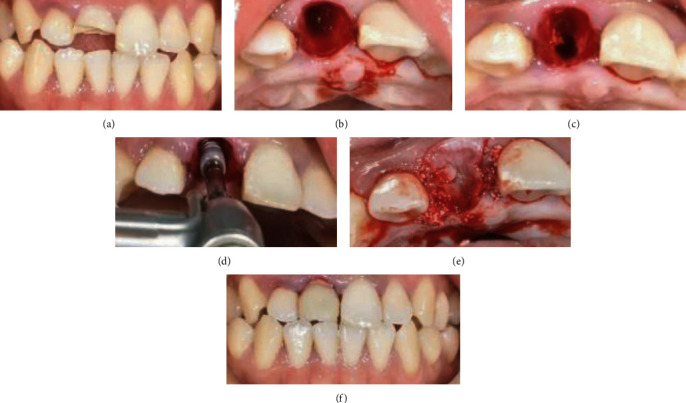
Immediate implant repair map. (a) The preoperative positive image. (b) After tooth extraction. (c) The backup cave map. (d) The implant. (e) Bone meal implanted and coated. (f) The postoperative positive view.

In the study group, immediate implant restoration was performed. After anesthesia took effect, the mucosa was cut along the extraction socket and the gingival was peeled off, and the edge of the extraction socket was fully exposed. According to the breadth of the tooth neck and the length of the root, the extraction socket was adequately extended. The autologous bone hole was collected at the standby hole when the implant's cervical margin reached 2 mm below the alveolar crest, and then, the bio-OSS shares were used for bone grafting and the Bio-Gide film was coated. The screw hole in the upper segment of the implant was screwed, and the mucosa was sutured, and the gauze was occlited for 30 minutes after surgery, as shown in Figure 1.

In the control group, a trapezoidal incision was made at 3 mm of the alveolar crest on the labial side about 5 weeks after tooth extraction, and transverse incision was made on the alveolar crest. Other steps were the same as those in the study group.

Both groups were inserted 2 weeks after surgery and followed up for 6 months.

#### 2.2.2. Data Acquisition and Digital Model Establishment

**Figure 2:**
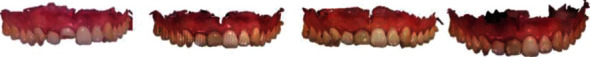
Oral scan models at different time points.

**Figure 3:**
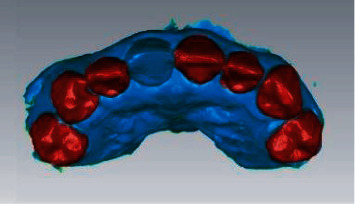
Note: the red area is the artificial overlap of similar surfaces of other teeth.

**Figure 4:**
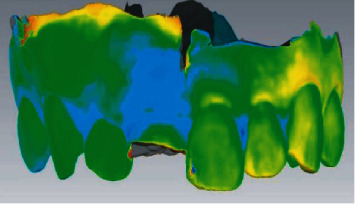
The overlap of preoperative and postoperative models and the change of soft tissue on the labial side at the implantation site by chromatography, in which the light blue area is the soft tissue collapse.

A professional physician performed preoperative and postoperative oral scans on the maxillary denture and labial gingival soft tissue to obtain digital impression modulus data (Figure 2), which will be entered into Geomagic Studio. The apical points with obvious features in the two models to be registered were selected, and the alignment function was performed, and the fitting alignment function was used for correction (Figure 3). The superimposed two models were analyzed by analytical function analysis, and the soft tissues around implants were labeled by 15-stage chromatography (Figure 4).

#### 2.2.3. 3D Reconstruction of Soft Tissue Morphology and 3D Morphological Measurement and Analysis

**Figure 5:**
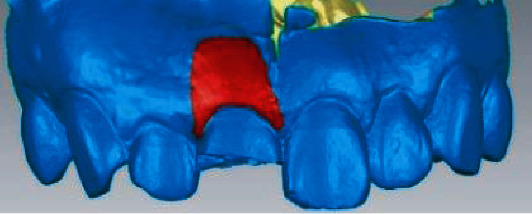
The changes of soft tissue at the implant site manually marked after 3D reconstruction.

**Figure 6:**
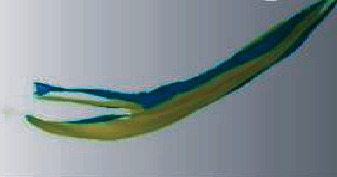
The flipped postoperative image, showing a gap between the two surfaces.

**Figure 7:**
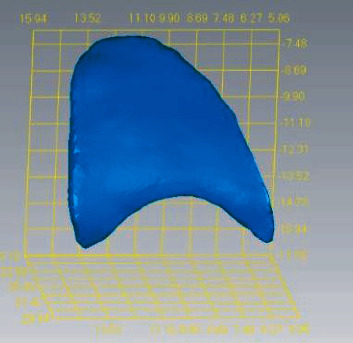
The three-dimensional morphology of soft tissue after reconstruction.

The preoperative scan model was superposed with the postoperative scan model, and the critical value MAX was set to 2.45 mm in the software, and the nominal values (MIX/MAX) were ±0.123 mm, respectively. When the change exceeded 0.123 mm, the color other than green would be displayed, so the aligned part was green and the soft tissue contour changed to blue in the model. And the darker the color, the higher the degree of change (Figure 4). Chromatography shows implant surrounding soft tissue changes (Figure 5) and keeps the green parts out the blue part and changes in the area of the surface of the two local choice of 6 months after surface and reverse flip; flip was observed before and after an internal space between two surfaces (Figure 6), the filled function for filling out this space so as to form a closed space. The reconstructed 3D model is the changes of soft tissues around the implant (Figure 7).

**Figure 8:**
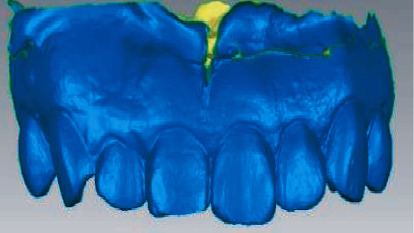
The integration of preoperative and postoperative linear measurements as a whole.

Professional surveyors will measure the superimposed model. The “Boolean Operation” function in the software will be used to integrate the superimposed model (Figure 8). Import the integrated model into Geomagic Qualify for section creation. The changes of 1 mm (RW1) and 3 mm (RW3) below the gingival mucosa margin were measured in the cross-sectional view again. The level of the baseline implant lip gingival (ML) was measured at the lip gingival apex of the preoperative end point, and the mean thickness of the soft tissue contour was measured (*D*).

#### 2.2.4. Aesthetic Evaluation of Pink in Planting Area (PES)

The evaluation scale mainly contains 7 items, including gingival color, texture, shape, alveolar ridge defect, gingival margin level, middle gingival papillary filling, and proximal middle gingival papillary filling. The score range is 0 to 14, and the lower the score is, the lower the aesthetic degree of the patient is.

#### 2.2.5. Evaluation of Implant Bone Absorption

The team completed in implant surgery and postoperative patients with six months for X-ray, stabilizing the tooth slice of filming process, and using image processing software for processing; after filming in a more accurate to evaluate implant absorption degree, the implant length is measured in the *X* line and compared the magnification (the length of the X-ray showed that planting-actual implant length), and the height of marginal alveolar bone was measured and bone resorption was evaluated. Those with bone resorption less than 1.5 mm were included in the excellent group, and those with bone resorption ≥ 1.5 mm were included in the poor group. The factors affecting bone resorption were analyzed by a single factor and multiple factors.

### 2.3. Statistical Treatment

The study data were put into SPSS 22.0 for statistical processing. If the measurement data followed normal distribution and homogeneity of variance, they were expressed as mean ± standard deviation. The independent sample *T* test was used to assess intergroup differences, while the paired *T* test was used to test intragroup comparisons. Counting data were represented by (%), and the differences between groups were tested by *x*^2^ test. Repeated measurement analysis and parallel spherical test were used for measurements at different time points. Univariate and multivariate analyses of factors affecting bone resorption of implants were done. All the above data were at *P* < 0.05, and the differences among the data were statistically significant.

## 3. Results

### 3.1. Postoperative Soft Tissue Changes in Each Group

The results showed that the levels of 1 mm and 3 mm below the gum mucosal margin in the two groups gradually increased over time after surgery, and the gingival level and soft tissue thickness at the lip of the baseline implant also gradually increased. However, the changes of soft tissue in the study group were better than those in the control group at 3 and 6 months after surgery (*P* < 0.05), as shown in Table 2.

### 3.2. Postoperative PES Score Changes in Each Group

PES scores of patients in both groups were significantly improved after treatment, and the aesthetic score of the study group was higher than that of the control group (*P* < 0.05), as shown in Table 3.

### 3.3. Univariate and Multivariate Analyses of Factors Affecting Bone Resorption of Implants

**Figure 9:**
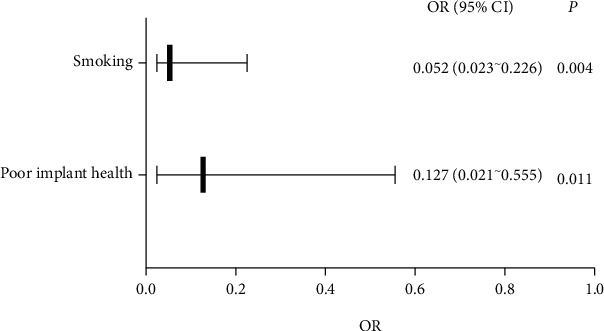
Multivariate analysis.

Univariate and binary logistic multifactor regression showed that smoking and poor implant health were the related factors affecting implant absorption (*P* < 0.05), as shown in Table 4 and Figure 9.

## 4. Discussion

As a treatment method for repairing the aesthetic area of the anterior teeth, immediate implant restoration of anterior teeth can better evaluate the clinical efficacy of this method for patients with tooth loss by observing the soft tissue changes after restoration [7]. In the past, worried models were commonly used in clinical practice to evaluate soft tissue changes, but this method was vulnerable to the influence of model materials and mould taking procedures, and the gypsum model measurement method was too solitary, leading in low accuracy of results [8]. With the continuous development of digital information technology, some scholars have pointed out that digital light scanning can be used to achieve multiangle measurement of the surrounding soft tissues of implants, so as to assist doctors to more accurately assess the prognosis of patients and provide corresponding treatment plans [9]. But at this stage on this technology is applied to the soft tissue changes after immediate implant prosthesis research is relatively small, therefore, this study through the line to our hospital were retrospectively analyzed to dental implant prosthesis of the 84 patients, compare the different repair methods on the influence of the soft tissue changes, and further analyze the related factors influencing the postoperative implant absorption, to provide clinical treatment basis for improving the clinical treatment of patients with anterior tooth loss.

The study's findings revealed that at 3 and 6 months following surgery, the level of 1 mm and 3 mm below the gingival mucosal margin, the level of baseline labial implants, and the thickness of soft tissue in the study group were higher than those in the control group (*P* < 0.05). By reviewing relevant literature and combining the results of this study, the author believed that, when making an immediate implant prosthesis because patients with tooth extraction socket are not yet healed, more effective orientation for judgment of alveolar socket must be done, so it is easier to implant into the ideal anatomical location, and more joint biomechanics, and immediate implant prosthesis can effectively shorten the patient's growing cycle, and reduce the missing tooth bone mass loss. It also plays a role in improving the quality of life [10]. Simultaneously, the changes in soft tissue generated by rapid implant repair could be caused by the restoration of the alveolar bone beneath the implant, leading in a change in alveolar crest size and then the retraction of the peripheral mucosa around the implant. In addition, studies have confirmed that there are many clinical factors that can cause the retraction of the peripheral mucosa. Based on this, some scholars have proposed that immediate implant repair can reduce postoperative mucosal edge retraction by reducing the degree of flap flap or avoiding flap flap [11, 12]. In addition, PES was used to compare the aesthetic degree of the two groups of patients after surgery. PES score of the study group was significantly higher than that of the control group (*P* < 0.05), which further demonstrated that immediate implant repair could not only promote the recovery of periodontal soft tissue but also enhance the aesthetic degree after surgery.

In addition, the risk factors causing poor implant absorption were analyzed in this study, and the results showed that postoperative smoking and poor implant health were independent risk factors affecting implant absorption (*P* < 0.05). Smoking is one of the factors causing poor implant absorption after surgery, which may be related to smoking inhibiting epithelial tissue generation and slowing down wound healing [13]. As a result, it is recommended that patients discontinue smoking prior to surgery and then reduce or quit smoking afterward, in order to ensure the greatest surgical outcomes and prognosis. In addition there are a large number of studies confirm that poor oral health situation is one of the important reasons influence a variety of oral disease, postoperative patients with immediate implant prosthesis implants with poor absorption also has close ties, and this study also showed that patients with poor oral health is another risk factor for postoperative implant absorption is poor, in line with previous studies [14, 15]. Therefore, it is recommended that patients keep their oral cavity clean after surgery, brush their teeth regularly to reduce and inhibit the formation of dental plaque, and improve the success rate of treatment and the effect of implant repair. Although this study has achieved some clinical results, due to the small sample size, the rigor of the results needs to be further supported by more scholars.

Finally, rapid implant restoration of anterior teeth can help the soft tissue around the implant recover more quickly and improve the overall aesthetic appearance. Meanwhile, reducing the frequency of smoking and maintaining clean oral hygiene after surgery can promote the implant restoration effect and clinical efficacy, improve the implant absorption degree, and further improve the prognosis.

## Figures and Tables

**Table 1:** Comparison of baseline data.

	Study group (*n* = 42)	Control group (*n* = 42)	*t*/*x*^2^	*P*
Age	35.23 ± 6.32	35.61 ± 6.29	-0.276	0.783
Gender			0.198	0.657
Man	26 (61.90%)	24 (57.14%)		
Woman	16 (38.10%)	18 (42.86%)		
BMI (kg/m^2^)	23.32 ± 2.14	23.21 ± 2.16	0.234	0.815
Tooth loss time (week)	5.58 ± 2.35	5.47 ± 2.42	0.211	0.833

**Table 2:** Postoperative soft tissue changes.

Group	Number		1 months after surgery	3 months after surgery	6 months after surgery	*F*	*P*
Study group	42	RW1 (mm)	0.14 ± 0.07	0.23 ± 0.12^∗^	0.28 ± 0.08^∗^^#^	2.214	0.023
		RW3 (mm)	0.21 ± 0.11	0.32 ± 0.19^∗^	0.39 ± 0.13^∗^^#^	3.342	0.038
		*D* (mm)	0.31 ± 0.11	0.52 ± 0.13^∗^	0.61 ± 0.16^∗^^#^	5.562	<0.001
		ML (mm)	0.22 ± 0.11	0.32 ± 0.18^∗^	0.41 ± 0.14^∗^^#^	3.267	0.026

Control group	42	RW1 (mm)	0.13 ± 0.08	0.22 ± 0.13^∗^	0.26 ± 0.09^∗^^#^	2.042	0.032
		RW3 (mm)	0.20 ± 0.12	0.30 ± 0.16^∗^^&^	0.35 ± 0.15^∗^^#&^	3.283	0.041
		*D* (mm)	0.28 ± 0.09	0.45 ± 0.14^∗^^&^	0.52 ± 0.15^∗^^#&^	4.983	<0.001
		ML (mm)	0.21 ± 0.06	0.27 ± 0.14^∗^^&^	0.35 ± 0.13^∗^^#&^	3.163	0.032

Note: ^∗^compared with 1 month after surgery, ∗*P* < 0.05, ^#^compared with 3 months after surgery, ^#^*P* < 0.05; and ^&^compared with the study group, ^&^*P* < 0.05.

**Table 3:** Aesthetic score changes after surgery.

Group	Number	Preoperative	6 months after surgery	*t*	*P*
Study group	42	6.63 ± 2.14	11.27 ± 2.31	-9.550	<0.001
Control group	42	6.52 ± 2.05	10.04 ± 2.16	-7.660	<0.001
*t*		0.241	2.521		
*P*		0.810	0.014		

**Table 4:** Univariate analysis.

	Good group (*n* = 26)	Bad group (*n* = 16)	*t*/*x*^2^	*P*
Age	36.23 ± 5.61	36.46 ± 5.66	-0.129	0.898
Gender			1.553	0.213
Man	18 (69.23%)	8 (50.00%)		
Woman	8 (30.77%)	8 (50.00%)		
BMI (kg/m^2^)	23.32 ± 2.10	23.21 ± 2.05	0.166	0.869
Smoking			17.374	<0.001
Y	3 (11.54%)	12 (75.00%)		
N	23 (88.46%)	4 (25.00%)		
Local health of implantation			4.423	<0.001
Cleaning	21 (80.77%)	5 (31.25%)		
Food residue	4 (15.38%)	5 (31.25%)		
Dental calculus	1 (3.85%)	6 (37.50%)		

## Data Availability

The datasets used in this paper are available from the corresponding author upon request.
